# Dysglycemia, glycemic variability and outcome after cardiac arrest and temperature management at 33 °C and 36 °C (a post-hoc analysis of the target temperature management trial)

**DOI:** 10.1186/2197-425X-3-S1-A843

**Published:** 2015-10-01

**Authors:** O Borgquist, MP Wise, N Nielsen, N Al-Subaie, J Cranshaw, T Cronberg, G Glover, C Hassager, J Kjaergaard, A Walden, H Friberg

**Affiliations:** Skåne University Hospital, Anesthesiology & Intensive Care, Lund, Sweden; University Hospital of Wales, Adult Critical Care, Cardiff, United Kingdom; Helsingborg Hospital, Anesthesiology and Intensive Care, Helsingborg, Sweden; St Georges NHS Hospital Trust, Cardiothoracic Intensive Care, London, United Kingdom; Royal Bournemouth Hospital, Intensive Care, Bournemouth, United Kingdom; Skåne University Hospital (Lund), Neurology, Lund Sweden; Guy's and St Thomas' NHS Foundation Trust, Critical Care, London, United Kingdom; Copenhagen University Hospital Rigshospitalet, Cardiology, Copenhagen Denmark; Royal Berkshire Hospital, Reading, United Kingdom; Skåne University Hospital (Lund), Anesthesiology & Intensive Care, Lund, Sweden

## Introduction

Dysglycemia and glycemic variability (GV) are associated with poor outcomes in critically ill patients. Target temperature management alters blood glucose (BG) homeostasis.

## Objectives

To investigate the association between BG levels and GV and the outcomes of patients randomized to target temperature management at 33 or 36 °C after cardiac arrest (CA).

## Methods

This is a post-hoc analysis of the TTM-trial [1], which included 950 patients with out-of-hospital CA of presumed cardiac cause at 36 sites in Europe and Australia from November 2010 to January 2013. A cerebral performance category of 1 or 2 was considered a favorable outcome. Patients who died during the first two days were excluded. Blood samples were collected and analysed per protocol at seven time points (0, 4, 12, 20, 28, 32 and 36 h). In addition, we included all registered BG values that were entered into the electronic case report form at different times between the fixed time points, at the discretion of the investigators. Four GV parameters (median, range, SD and MAG) were used.

## Results

**Figure 1:**
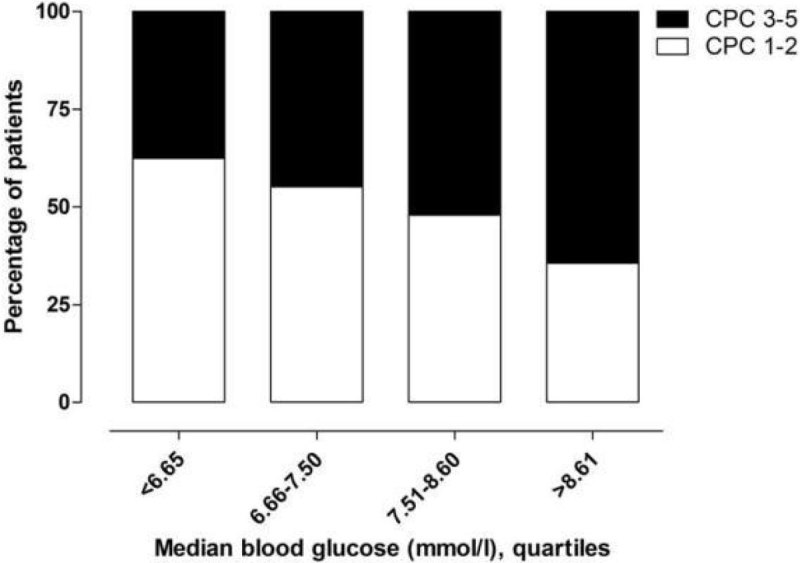
Figure 1

874 patients, 437 in each of the temperature groups, were included. Overall, 479 (55%) patients survived and 440 (50%) had a favorable neurological outcome. Patients with a favorable outcome both had a lower median BG level at admission (10.1 mmol/L vs 11.7 mmol/L, *p* < 0.001) and lower median BG levels during the first 36 hours compared to patients with unfavorable neurologic outcomes (fig 1). Hyperglycemia (>10 mmol/L) was more common among patients with a poor outcome, and hypoglycemia ( < 4 mmol/L) was associated with mortality. The incidence of hypo- and hyperglycemia did not differ between the two temperature groups. Overall, 52% of the patients received insulin treatment, 230 (55%) in the 33 °C group *vs* 197 (48%) in the 36 °C group (*p* = 0.04). Fewer patients with a good neurological recovery received insulin treatment as compared to those with a poor neurological outcome (181 [43%] *vs* 250 [60%], *p* < 0.001).

In unadjusted analysis, all GV parameters were lower among patients with a good outcome. In multivariate analysis, range was a predictor of survival (*p* = 0.047, OR 1.04 [1.00-1.09]) whereas median neither proved to be an independent predictor of neurological outcome (*p* = 0.15) nor of survival (*p* = 0.076). Range and SD were higher in the 33 °C group, whereas median and MAG did not differ between the temperature groups.

## Conclusions

Patients with a favorable outcome had lower median BG levels on admission, lower incidence of hyperglycemia and required less insulin treatment while GV did not differ between those with favorable and poor outcome. There was no difference in dysglycemia incidence between 33 °C and 36 °C, but more patients needed insulin treatment and GV was higher with a target temperature of 33 °C.
